# Dynamic Characteristics Study for Surface Composite of AMMNCs Matrix Fabricated by Friction Stir Process

**DOI:** 10.3390/ma11071240

**Published:** 2018-07-19

**Authors:** Essam B. Moustafa

**Affiliations:** Mechanical Engineering Department, Faculty of Engineering, King Abdulaziz University, Jeddah 21589, Saudi Arabia; abmostafa@kau.edu.sa; Tel.: +966-54-0886498

**Keywords:** FSP, dynamic modeling, dynamic characteristics, modal analysis, natural frequency, FRF, AMMSNCs

## Abstract

In the present work, Aluminum Metal Matrix Surface Nano Composites (AMMSNCs) were manufactured using Friction Stir Processing (FSP). Moreover, the fabricated surface composite matrix was exposed to a different number of tool passes with different processing parameters. The tensile test and microstructure examinations were used to study the mechanical properties of the composite surface. The dynamic properties were predicted using modal analysis and finite element methods. After this, dynamic characterization was achieved by combining the numerical and experimental methods to investigate the effects of changing the number of passes on the natural frequency and the damping capacity of the AMMSNCs manufactured using FSP. The results indicated that the damping capacity and dynamic behavior improved with an increased number of FSP passes.

## 1. Introduction

Applications that are subject to dynamic effects, especially in aircraft and vehicles, need materials with the highest possible damping coefficients and the best possible mechanical properties. In the last few years, Friction Stir Welding (FSW) and its development technique, Friction Stir Processing (FSP), have been widely used in industry. Furthermore, FSP is developed on the basic principles of FSW and aims to further improve the mechanical properties. In this method, a rotating tool with a pin and shoulder is fed to the workpiece, which causes intensive plastic deformation and mixes the material at higher temperatures. This leads to an increase in the homogeneity of the processed zone and refinement of the microstructure. The tool rotation speed, tool feed speed, and the number of tool passes are the main parameters that affect the mechanical behavior [1,2]. Furthermore, the addition of reinforcements in the form of nanoparticles, which come in different types and sizes, have enhanced the mechanical properties. Aluminum alloys have been developed and used in various industries due to their lower density and good strength with respect to weight and corrosion resistance. Metal matrix composites (MMCs) are new materials with excellent mechanical tribological properties [3].

The processing parameters, such as tool rotation speed, feed, and number of passes, play an important role in determining the surface composites. Moreover, the type and size of the nanoparticles used for reinforcement will affect the mechanical properties. The mechanical properties can be improved significantly by adding the reinforcement nanoparticles into the surface layer using an FSP technique. This investigation shows that a greater increase in the rotation speed has a greater effect on the surface layer thickness and grain size. Furthermore, this increase causes better dispersion and distribution of nanoparticles in the surface layer of aluminum alloys [4,5]. Nakata et al. [6] used multi-pass FSP and different types of reinforcement nanoparticles to increase the tensile strength of AMMNCs. However, these reinforcement particles decrease the ductility and enhance the tensile strength and other mechanical properties [7]. The homogenous distribution of these nanoparticles over the processed zone improves the wear resistance during FSP [8]. From another perspective, the multiple tool passes of FSP results in a decrease in the grain size, which causes an improvement in yield and ultimate tensile strength. However, it results in a decrease in the porosity of the contents [9,10,11]. Consequently, aluminum matrix that was reinforced with nanoparticles using FSP has improved mechanical properties in term of hardness and tensile strength.

Dynamic behaviors of the composite materials and structures were investigated in order to improve the damping behavior at high temperatures [12,13,14]. Many authors have investigated dynamic characteristics of composite materials and structures using free vibration methods [15,16,17,18,19,20]. Adding reinforcement particles to the metal matrix composites can change their mechanical and dynamic properties. The effect of thermal stresses on the damping capacity of aluminum metal matrix composites was investigated by reference [21]. The results showed that the damping capacity of the metal matrix composites improved with an increase in temperature during the heating process. Combined finite element and frequency response was carried out by references [22,23], who aimed to study the dynamic properties of the composite material. The study of the damping properties and modelling of the dynamic properties have been conducted by many investigators [24,25,26,27,28] using the finite element methods to predict the natural frequency and mode shapes. These results allow us to characterize the dynamic behaviors for metals and new composite materials.

The present study is a continuation of a previous study by Moustafa [29], which focused on the effects of processing parameters on the mechanical properties, such as microstructure, micro-hardness, and tensile properties. In the current work, the dynamic characteristics of the FSP along the processing pathway were investigated using experimental and mathematical simulation methods. The complex or dynamic moduli were determined by the nondestructive free vibration technique, while static Young’s moduli were calculated using the classic tension test. A parametric study and modal analysis were performed using finite element ANSYS 17 software in order to predict the dynamic characteristics of the AMMNSCs. Furthermore, the effects of processing parameters on the damping capacity were verified using both the experimental results of natural frequency and the results from the simulated model. 

## 2. Experimental Procedure

**Figure 1:**
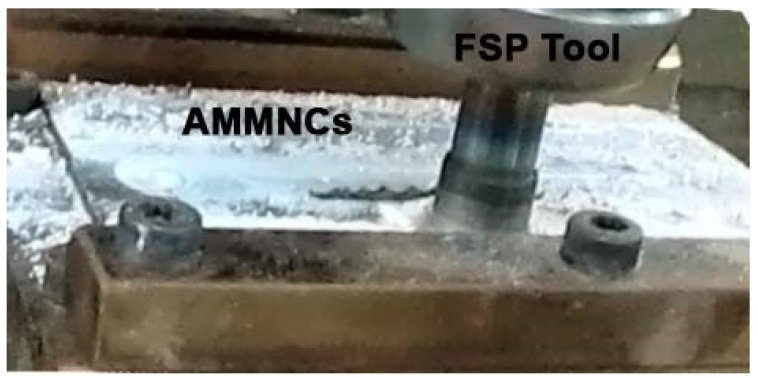
Friction Stir Processing (FSP) using vertical milling machine with fixture installation.

Multi-pass FSP allows us to refine the grain size of Al, which consequently improves the mechanical properties. AA 2024 alloy was used as the base alloy for performing FSP. Aluminum alloy plates were prepared and machined in order to have a suitable size for processing. The plates were grooved longitudinally by an end mill tool, with each groove having a diameter of 3 mm and a depth of 2 mm. These grooves were filled with Al_2_O_3_ nano-particles with an average diameter of 30 nm in order to reinforce the Al metal matrix. The friction stir processing tool was manufactured from hardened K-110 tool steel. The FS tools were machined to have cylindrical geometry with a pin of (Ø8) mm and a shoulder of (Ø25) mm in diameter, while the tool pin length was 3.5 mm. The processing was performed using an automatic milling machine (Bridgeport, Elmira, NY, USA) as shown in Figure 1. The main processing parameters used in this study include different numbers of FSP passes, different tool rotation speeds from 1000 rpm to 2000 rpm, and three traverse speeds of 10, 15 and 20 mm/min. The tensile test was carried out according to ASTM B557 for aluminum alloys in order to calculate the engineering Young’s modulus. All samples were cut in a direction that was parallel to FSP. The engineering Young’s modulus and other mechanical properties were calculated. In order to study the shape of particles and microstructural characteristics of FSP, the specimens were examined using an optical metallurgical microscope and scanning electron microscopy (SEM, Quanta 250 FEG, Hillsboro, AL, USA). 

## 3. Mechanical Properties

**Figure 2:**
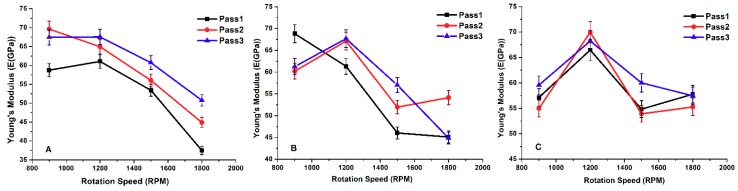
Effect of the number of passes on Young’s Modulus at (**A**) 10 mm/min travel speed; (**B**) 15 mm/min travel speed and (**C**) 20 mm/min travel speed.

In the previous study [29], the tension test was performed in order to calculate the composite matrix mechanical properties, such as Ultimate Tensile Strength “UTS”, Yield Strength “YS”, and Young’s modulus “*E*”. Figure 2 shows the effect of pass number on Young’s modulus under different processing conditions. The results revealed that the mechanical properties improved when the number of FSP passes increased.

From all the previous experimental data, a direct relationship was established to estimate the value of Young’s modulus (Equation (1)). This new equation is expressed as a multi-variable power equation, which correlates Young’s Modulus “*E*” to the number of passes “*P*”, tool rotation speed “*S*” in rpm, and the tool traverse speed “*V*” in mm/min. A curve fitting was used to estimate the values of the power factor with an error of less than 10%.

(1) $$E=470.09P^{0.0849}S^{-0.33}V^{0.0766},$$

## 4. Dynamic Characterization

In this section, dynamic characteristic was achieved by combining the numerical and experimental methods to study the effect of changing the number of passes on the natural frequency, mode shape, and the damping of the FSP and AMMSNCs.

## 5. Finite Element Model (FEM)

Finite element analysis was developed as a very efficient tool for solving complex problems in the field of design engineering. Many authors [24,30,31,32] used FEM to model the cantilever beam. In this paper, a finite element model was developed to simulate both partially surface composite and fully surface composites. The mode shapes and frequency response function (FRF) have been determined. The AMMSNCs have already been modeled as a cantilever beam. Hence, the model was used to determine a structure’s vibration characteristics, natural frequency, and mode shapes. In this model, the modal analysis module was selected to perform the dynamic study. Moreover, the harmonic response module was used along with modal analysis to predict the frequency response function (FRF). 

**Figure 3:**
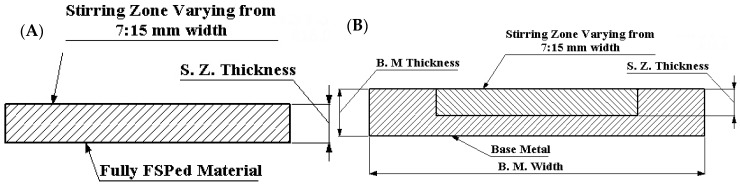
Two different modeled geometries: (**A**) homogeneous rectangle beam; (**B**) cross section in the surface composite rectangle beam.

A parametric study was performed on the beams with different geometry parameters. Variations in the length, width, and thickness were considered in the study. Furthermore, the AMMNSC properties were selected based on the previous test of mechanical properties. Increasing the number of FSP passes changes the mechanical properties. Therefore, Young’s modulus and Poisson's ratio were calculated based on the mechanical properties obtained from experimental results in order to be used as a material input parameter for the model. The used geometries are shown in Figure 3A. The homogeneous rectangle beam represents the fully surface composite beam, which can be modelled as a base metal (Figure 3). Furthermore, the partially surface composite beam with FSP surface is shown in the diagram. 

**Figure 4:**
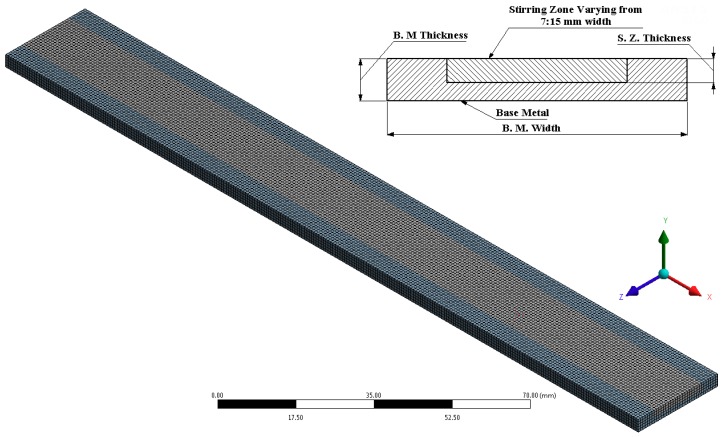
Meshing methods for surface composite processed beam.

A mesh study was conducted to select the most appropriate size and type of mesh. After converging the output data error, the mesh size and type were determined. It was observed that the hexahedrons meshing type is the most suitable type as shown in Figure 4. Moreover, the mesh density was found to have a length of 180 elements and thickness of 4 elements. This hypothesis was created to allow for reasonable frequency calculations. After this, a modal shape analysis was performed to obtain the natural frequencies for the two models of the fully and partially (surface composite) FSP. Harmonic analysis was performed by entering the frequency range (0–7000 Hz) according to the frequency range calculated from the first step in modal analysis and mode shape frequency. After this, the boundary conditions were applied by providing a force at the other end of the cantilever beam. A nondestructive vibration hammer test technique was used as previously described by many researchers [33,34,35] in order to characterize the dynamic properties of the fabricated materials. 

**Figure 5:**
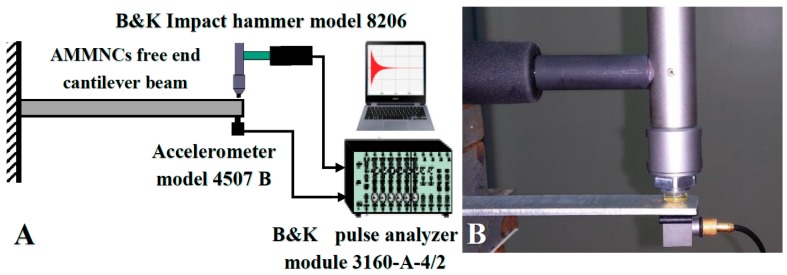
(**A**) Schematic diagram for the experimental setup of free vibration tests; (**B**) typical test rig and the equipment used in free vibration test.

**Figure 6:**
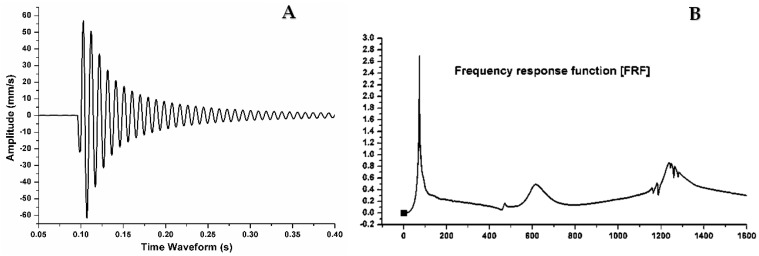
(**A**) Experimental output time domain; (**B**) Frequency domain using Fast Fourier Transformation (FFT) analysis.

In the present work, an experimental free vibration test was performed on the (AMMSNCs) beam to identify the damping factor and natural frequency. Rectangle beams with 90-mm length, 15-mm width, and 2-mm thickness were used in the test. The specimen was prepared as a cantilever beam with one free end. The time decay was measured using an accelerometer (B&K model 4507 B) mounted to the free end of the cantilever beam of AMMNSCs. The beam was excited by an impact hammer (B&K model 8206, Brüel & Kjær, Nærum, Denmark). The vibration response was measured and analyzed using a pulse data analyzer (B&K module 3160-A-4/2 Brüel & Kjær, Nærum, Denmark). Figure 5 shows the experimental test rig setup used in the study. The frequency response function (FRF), damping ratio and fundamental frequencies were calculated using modal analysis software (ME Scope) as shown in Figure 6. The free vibration test was carried out and repeated seven times in order to obtain an accurate value.

## 6. Results of Finite Element Model

**Figure 7:**
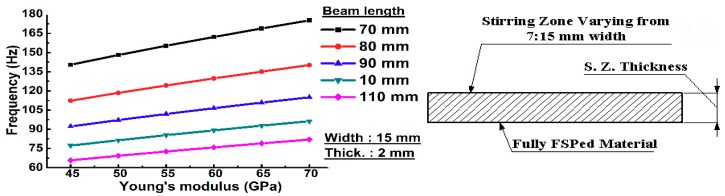
Effect of geometry on the dynamic properties for the fully composite beam, which was determined in a parametric study.

**Figure 8:**
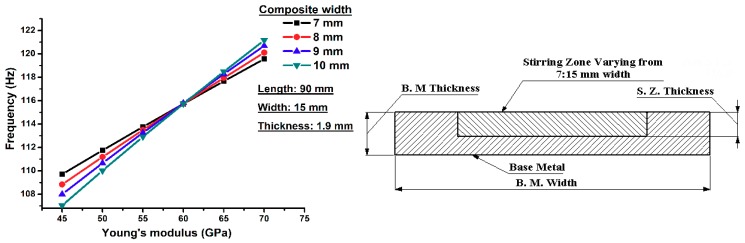
Effect of the width of the composite surface on the dynamic response with variable Young’s modulus.

A parametric study is considered to be an effective tool to obtain a set of variables and parameters without changing the model setup. Four major parameters are used in this investigation in order to study the influence of material geometry and properties on the dynamic behavior, including: effect of length, width, thickness and Young’s modulus of the resultant frequencies. There is an inverse relationship between length and the natural frequency as shown in Figure 7. We used a fully composite beam in the simulated model. Different values for Young’s modulus was used in order to simulate the fabricated samples. The parametric study demonstrated that the natural frequency has direct relationship with the width and thickness. The surface composite beam was modeled in a similar way to the previous study. Figure 8 shows the effect of composite volume parameters on the natural frequency when using different Young’s moduli. In addition, the base metal has a constant Young’s modulus of 60 GPa. An increased volume of the composite matrix results in an increased natural frequency of the overall surface composite structure. In this model, when the composite surface layer has poorer mechanical properties than the base material, there is an inverse relationship between the width of the composite layer and dynamic frequency response. When the composite layers have better mechanical properties than the base metal, there is a direct relationship between Young’s modulus and natural frequency.

## 7. Modal Analysis Results 

**Figure 9:**
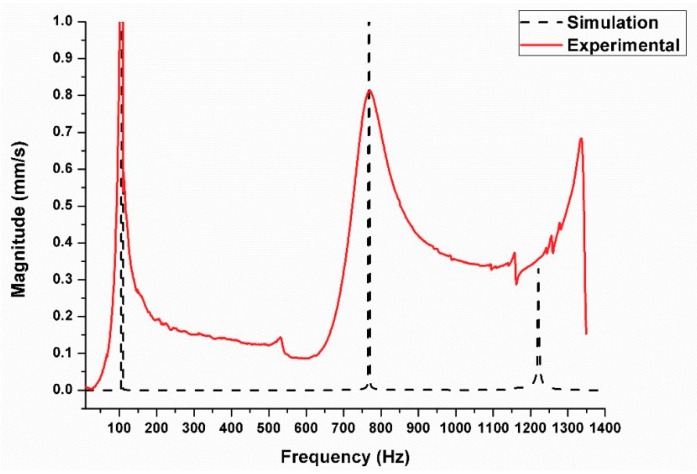
Frequency response function (FRF) comparison between the experimental free vibration test and the simulation modal for the surface composite beam with an average Young’s modulus of 65 GPa.

In this study, the different mode shapes and their corresponding natural frequencies were simulated. Referring to the data given in the previous sections, the three models were simulated in order to obtain the fundamental frequencies in each case. The results are summarized in Table 1. The results reveal that there is no significant difference in the values of natural frequency. In particular, this lack of significant differences occurs in the first three-mode shape and their corresponding frequencies. The frequency response function (FRF) for both the simulated model and experimental free vibration test are illustrated in Figure 9. 

## 8. Results of Dynamic Properties. 

In the current work, the free experimental vibration tests were performed on the surface composite beam. The pulse impact method was applied to identify the resonant frequency in each sample. The modal analysis software (Structural Vibration Solutions, Aalborg East, Denmark) computes the dynamic parameters obtained from the time domain curves.

The decay curve method and Fast Fourier Transformation (FFT) analysis were used to calculate the damping ratio (*ζ*) and frequency response function, respectively. The damping ratio was measured by the vibration accelerometer (Brüel & Kjær, Nærum, Denmark) as a function of time. The damping ratio was obtained by Equations (2) or (3) [34] or by using the modal analysis software. The storage modulus, complex modulus of elasticity and loss factor were calculated according to Equations (4)–(6) [35,36,37]. The results, which are shown in Table 2, demonstrate the dynamic properties of the surface composite beam with respect to the base aluminum alloy. In addition, we observed variation in the dynamic characteristics between the as-received alloy (AA2024) and the composite surface fabricated by multi-pass FSP.

(2) $$\delta =\frac{1}{n}ln\frac{x^{0}}{x_{n}},$$

(3) $$\zeta =\sqrt{\frac{\delta^{2}}{\delta^{2}+4\pi^{2}}},$$

(4) $$E^{\prime }=\frac{4\pi^{2}f^{2}}{3I}\times \left[M+\frac{33}{140}m\right]\times L^{3}\times \left[1+\frac{\delta^{2}}{4\pi^{2}}\right],$$

(5) $$E^{*}=E^{\prime }+jE\prime \prime ,$$

(6) $$\eta =\frac{E^{\prime }}{E^{\prime \prime }}=\tan\left(2\zeta \right),$$

where *M* is the mass of the AMMNSCs cantilever beam (kg), *m* is the mass of the accelerometer, *L* is the free beam length (m), *I* is the area moment of inertia (m4), *fn* is the first mode natural frequency, *δ* is the logarithmic decrement, E* is the complex modulus of elasticity, *E′* is the elastic (or storage) modulus, *E**″*** is the damping (or loss) modulus, and $\left[j\right]=\sqrt{-1}$.

## 9. Effect of Linear Travel Speed on the Damping Capacity

**Figure 10:**
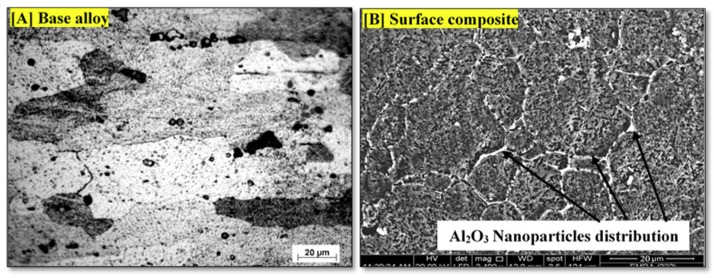
(**A**) Microstructure of 2024 base alloy; and (**B**) Al_2_O_3_ nanoparticles dispersion in the surface composite fabricated by FSP.

The results show that the linear travel speed has a significant effect on the damping capacity when fabricating the surface composite using FSP. Figure 10A shows the microstructure of the as-received AA2024 alloy, which has a larger grain size with intermetallic components. After having applied FSP to the surface of the matrix, the grains become finer and the Al_2_O_3_ nanoparticles are distributed homogenously around the boundary of the grains as shown in Figure 10B. Adding alumina nanoparticles to the mixture enhances the damping capacity of the fabricated composite. 

**Figure 11:**
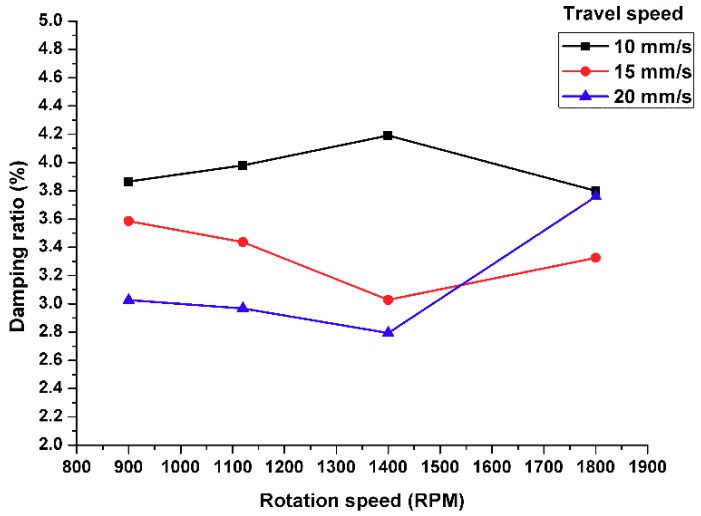
Effect of travel speed on the damping capacity.

A low feed rate increases the heat generated during the process, which allows Al_2_O_3_ nanoparticles to redistribute around the grain boundary of the base metal microstructure. Furthermore, it decreases the microstructure grain size as shown in Figure 11. These results are consistent with previous studies [38,39]. 

The damping capacity was enhanced at a relatively lower rotation speed, while higher travel speeds and tool rotation speeds did not improve the damping values.

## 10. Effect of the Number of FSP Passes on the Damping Capacity

**Figure 12:**
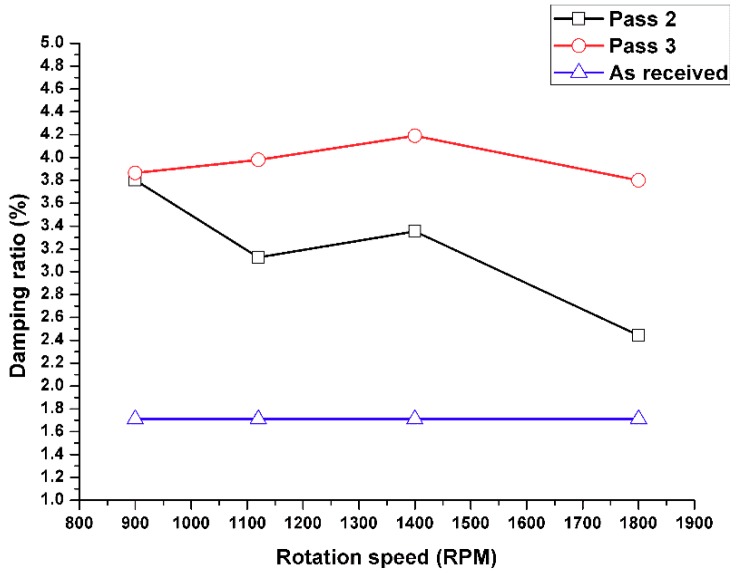
Effect of multi-pass FSP on the damping capacity of the surface composite matrix at a tool rotation speed of 1400 rpm and feed rate of 10 mm/min.

Multi-pass FSP is considered to be one of the most important processing parameters as it can improve both the mechanical properties and dynamic properties. A better distribution of the Al_2_O_3_ nanoparticles and improved microstructure of the grains can improve the damping capacity of the processed metal matrix nanocomposites. Figure 12 shows the effect of the number of FSP passes as three FSP passes were applied in this present study. The first pass was excluded due to the incomplete fabrication of the surface composite matrix. Thus, the combination matrix of aluminum alloy and dry Al_2_O_3_ nanoparticles need more than one pass of FSP to obtain the required fabricated surface composite metal matrix. The as-received alloy demonstrated a lower damping capacity after the second and third FSP passes. 

**Figure 13:**
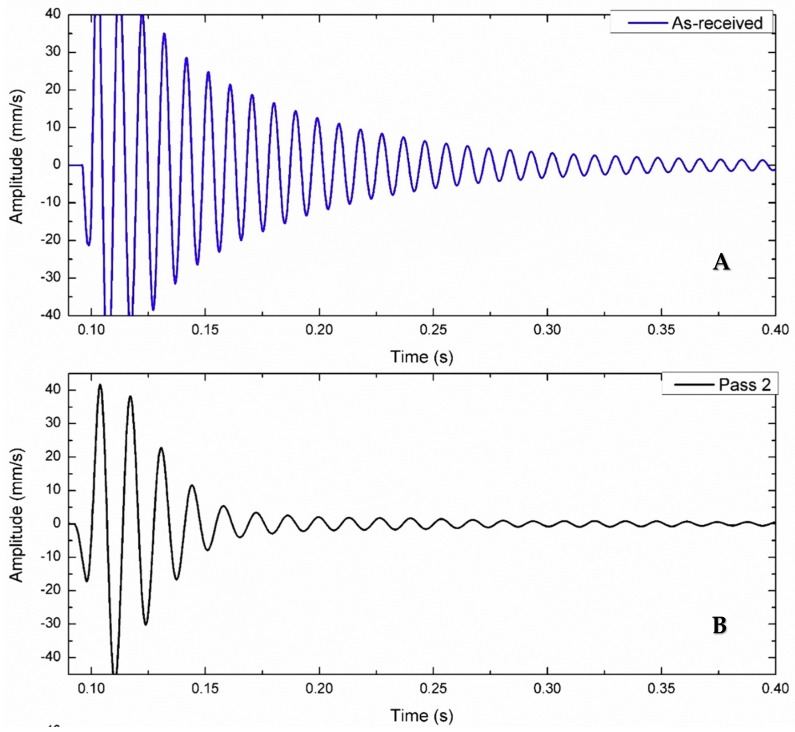

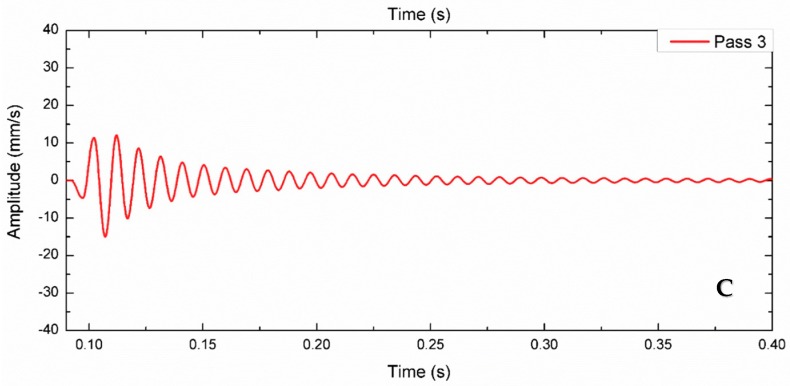
Effect of FSP multi-pass on damping capacity using the time decay stack curve at a tool rotation speed of 1400 rpm and a feed rate of 10 mm/min: (**A**) as received alloy; (**B**) Second pass FSP; (**C**) Third pass FSP.

Figure 13 shows the time decay response for the as-received alloy after the second and third FSP passes. There are significant variations in the decay time between the base alloy and the other processed samples. In the first curve, the time wave was damped after 0.4 s, while the time wave was damped in 0.25 s in the third curve. Moreover, the number of decayed cycles was reduced by 70%.

## 11. Verification of the Dynamic Properties

**Figure 14:**
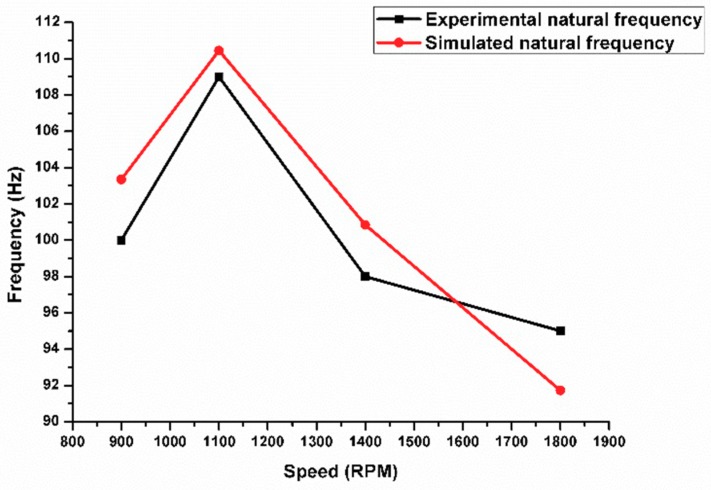
Measured natural frequency values against predicted frequency using modal analysis simulation.

**Figure 15:**
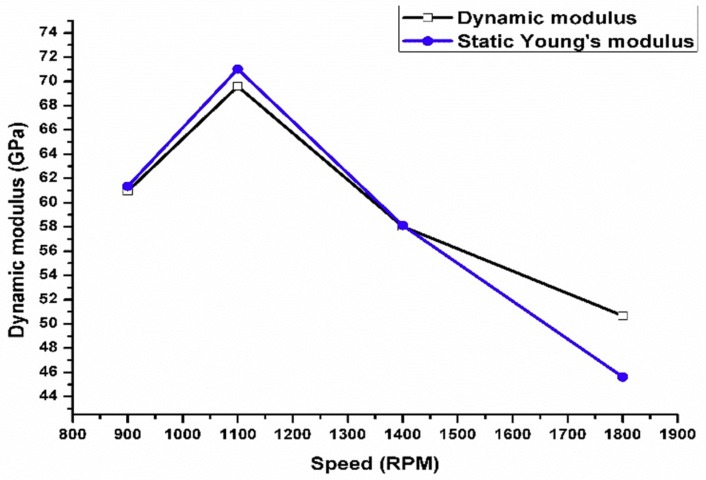
Convergence of static and dynamic moduli.

The finite element model was verified with the experimental natural frequency obtained from the free vibration test. Figure 14 shows a significant convergence between the experimental natural frequency and simulated frequency with an average error of 5%. The natural frequency calculated from the experimental free vibration test was used in Equation 4 in order to calculate the dynamic or (complex) modulus. The static engineering Young’s modulus was obtained from a tension test according to ASTM B557. The samples were cut in a direction that was parallel to FSP. The engineering Young’s modulus was calculated from the applied tension load and sample elongation value. The results revealed that there is a significant convergence between the experimental engineering Young’s modulus and that obtained from the nondestructive experimental test for dynamic moduli with an average error of 3.5%. These values are close, especially at a lower tool rotation speed (Figure 15). 

## 12. Conclusions 

From the current investigation, the following conclusions are drawn:

- The natural frequencies obtained by the simulation model were close to the values obtained from the experimental free vibration test.
- The damping capacity for the surface composite beam was enhanced with respect to the base alloy by 44% as it acts as a self-damping material. This is due to the presence of Al_2_O_3_ nanoparticles, which are homogenously dispersed in the metal matrix. Furthermore, this damping capacity can be improved by using an increased number of FSP passes.
- A significant improvement in the damping ratio was obtained from the third pass of FSP.
- The dynamic properties were enhanced at a lower feed rate speed, with the optimum values observed at a travel speed of 10 mm/min. This can be explained by the fact that a lower travel speed allows for adequate heating time and homogenous distribution of nanoparticles in the surface composite matrix.
- The results revealed that there is consistency between the dynamic and static engineering Young’s moduli.

## Figures and Tables

**Table 1:** Three mode shapes with corresponding fundamental frequencies in the simulation model and experimental test.

Mode	Simulation Model			Experimental Result
	Fully Composite (Hz)	Surface Composite (Hz)	Base Material (Hz)	Surface Composite (Hz)
1.	110.6	108.34	102.55	104
2.	802.17	772.85	743.78	762
3.	1260.7	1225.6	1168.9	1335.9

**Table 2:** Dynamic properties of the investigated surface composite beam at a constant processing parameter travel speed of 10 mm/min.

Speed RPM	Natural Frequency (Hz)	Damping Ratio (ζ)	Loss Factor (η)	Storage Modulus (E′) GPa	Loss Modulus (E″)	Complex Modulus (E*) GPa	Shear Modulus (G) GPa	Number of Passes	Static Young’s Modulus GPa
Base alloy	101	1.71	0.0342	61.754412	2.1120009	61.790517	23.25354	-	59.94788221
900	100	3.85867	0.077173	60.7621471	4.689221	60.94282	22.84291	3	61.3297661
1100	109	4.09065	0.081813	69.45885	4.34257	69.59447	26.11235	3	71.01231
1400	98	4.19065	0.08381	57.81953	4.846028	58.02225	21.73667	3	58.1162103
1800	95	3.4175	0.06835	50.5553034	3.455455	50.67326	19.00575	3	45.61504421
